# High quality thin films of thermoelectric misfit cobalt oxides prepared by a chemical solution method

**DOI:** 10.1038/srep11889

**Published:** 2015-07-08

**Authors:** Beatriz Rivas-Murias, José Manuel Vila-Fungueiriño, Francisco Rivadulla

**Affiliations:** 1 CIQUS, Center for Research in Biological Chemistry and Molecular Materials. University of Santiago de Compostela, c/Jenaro de la Fuente, s/n, Campus Vida, 15782-Santiago de Compostela, Spain

## Abstract

Misfit cobaltates ([Bi/Ba/Sr/Ca/CoO]_n_^RS^[CoO2]_q_) constitute the most promising family of thermoelectric oxides for high temperature energy harvesting. However, their complex structure and chemical composition makes extremely challenging their deposition by high-vacuum physical techniques. Therefore, many of them have not been prepared as thin films until now. Here we report the synthesis of high-quality epitaxial thin films of the most representative members of this family of compounds by a water-based chemical solution deposition method. The films show an exceptional crystalline quality, with an electrical conductivity and thermopower comparable to single crystals. These properties are linked to the epitaxial matching of the rock-salt layers of the structure to the substrate, producing clean interfaces free of amorphous phases. This is an important step forward for the integration of these materials with complementary n-type thermoelectric oxides in multilayer nanostructures.

Layered misfit cobalt oxides of the type [AO]_n_^RS^ [CoO_2_]_q_ (A = Ba, Ca, Sr, Bi, Co) are structurally and chemically complex materials, in which conducting planes of edge-sharing CoO_6_ octahedra are stacked along the c-axis within insulating layers of rock salt structure (RS)^1^,^2^. These building blocks have the same a- and c-lattice parameters, but differ in the b axis (b_1_ and b_2_ are the parameters of the RS and the CoO_2_ layers, respectively; q = b_1_/b_2_)^3^,^4^,^5^. Many different compositions of this family of materials have been synthesized, with either n = 3 or n = 4-pseudoquadratic layers in the RS block. Among them, the 3-layer [(Ca,Sr)_2_CoO_3_]^RS^ [CoO_2_]_q_ (referred hereafter as [CaCo] and [SrCo]) and the 4-layer [Bi_2_(Ca,Sr,Ba)_2_O_4_]^RS^ [CoO_2_]_q_ (referred hereafter as [BiCaCo], [BiSrCo], and [BiBaCo]) are the most studied, because they constitute one of the few examples of p-type oxides with good thermoelectric (TE) properties^6^,^7^,^8^,^9^.

The synthesis of thin-films of these oxides is highly desirable for their integration in different platforms for advanced “on-chip” applications and fabrication of TE modules. However, their deposition by high-vacuum physical methods is challenging, due to the difficulty of controlling a stoichiometry transfer of such multi-cation structures, and many of these complex oxides (particularly the n = 4) have not been prepared as thin films yet.

Some examples of [AO]_n_^RS^ [CoO_2_]_p_ (A = Ba, Ca, Sr, Bi, Co) thin films fabricated by sputtering^10^,^11^, pulsed laser deposition (PLD)^12^,^13^,^14^,^15^, and molecular beam epitaxy^16^ are reported in the literature, but their transport properties are extremely dependent on the deposition conditions. Moreover, in many of these cases an amorphous interface layer is formed close to the substrate^17^,^18^, which impedes their integration in a functional multilayer device.

On the other hand, Bi_2_Sr_2_Co_2_O_4_ and Ca_3_Co_4_O_9+δ_ were fabricated by sol-gel chemical methods^19^,^20^,^21^. But in many cases the structural quality of the films is poorer than achieved by vacuum physical deposition, and a single chemical method suitable for the fabrication of all the 3 and 4-layer compounds is still lacking, which limits the applicability and recognition of these techniques.

Therefore, it is imperative to develop alternative strategies to produce stable, highly crystalline thin films of thermoelectric misfit oxides that allow their integration in multilayered modules. Here we report the chemical solution synthesis of high-quality epitaxial thin films of all the representative members of this family of compounds, including the complex 4-layer compounds. The films show an an electrical conductivity and thermoelectric power comparable to single crystals. We demonstrate that the quasi-equilibrium growth characteristic of this method favors the epitaxial matching between the RS lattice and the substrate, therefore promoting a well ordered self-assembled growth along the thickness of the film, free of amorphous interfaces. The origin of the large thermoelectric behavior of misfit cobalt oxides is discussed at the end of the paper.

## Experimental Section

Dealing with such complex solutions, involving a large number of different cations with diverse reactivity is not an easy task: undesired cross-reactions, or non homogeneous precipitation of the oxides is a typical problem in chemical-solution methods. To overcome these difficulties, we have used water-based solutions in which the different cations are coordinated to ethylenediaminetetraacetic acid (EDTA) and polyethileneimine (PEI). Individual solutions of the different metal ions were prepared by dissolving the corresponding nitrate salts in water, and EDTA (1:1 molar ratio). PEI was incorporated to the solution in a 1:1 mass ratio to EDTA. Each individual solution was filtrated using Amicon units (10 kDalton), and retained portions were analyzed by Inductively Coupled Plasma (ICP). The final concentration of the solutions used in this work were [Bi] = 79.2 mM, [Co] = 89.8 mM, [Ca] = 107.2 mM, [Sr] = 138.4 mM, and [Ba] = 89.1 mM. These solutions were mixed according to the desired final stoichiometry, and concentrated to reach a final cation concentration of ≈200 mM. These conditions were adjusted to produce films in the range of ≈20–30 nm. Cross-section TEM of the films, as well as top-view AFM and SEM images are shown in Figures S1 and S2 in the supporting information.

The final solutions are then spun-coated on (001) LaAlO3 (LAO) substrates, and annealed at 750 °C for 2 h in air with both slow heating and cooling ramps. PEI decomposes at ≈575 °C, right before the crystallization of the inorganic film^22^. This stabilizes the cations and prevents any other undesired reaction to occur. Taking advantage of the quasi-equilibrium growth conditions of this method we successfully grew the elusive 4-layer [BiCaCo], [BiSrCo], and [BiBaCo] phases, with an exceptional crystalline quality. Thin films of the 3-layer [CaCo] and metastable [SrCo] were also successfully synthesized.

The electrical resistivity and Hall coefficient was measured in a Van der Pauw geometry, using Al wires directly bonded to the sample. For measuring the Seebeck coefficient two Ag strips were evaporated on the sample, and a thermocouple (in a differential configuration) was attached to them. The values of ΔT and ΔV were recorded, at each base temperature, as a function of the power dissipated by a ceramic heater attached to one end of the sample. The linear fitting of the ΔS vs ΔT gives directly the thermoelectric power at each temperature.

## Results and Discussion

**Figure 1:**
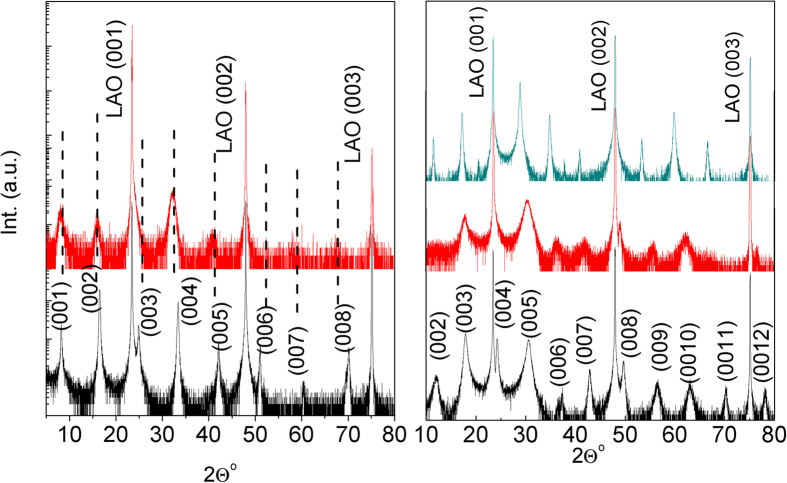
X-ray diffraction patterns of the different samples synthesized in this work. The patterns of the 3-layer [CaCo] and [SrCo] (bottom to top) are shown in **a**). The vertical dotted lines mark the position of the less intense peaks in the [SrCo] phase. In **b**) are shown the results for the 4-layer: [BiCaCo], [BiSrCo] and [BiBaCo] (bottom to top).

The X-ray diffraction (XRD) patterns are shown in Fig. 1 for all the samples deposited in this work. The films are deposited on [001]-oriented LaAlO_3_ (LAO) substrates. Only the (0 0 l) reflections are observed, indicating that the films are single phase and perfectly aligned along the c-axis. The out-of-plane lattice parameters are 10.73(1) Å and 11.10(1) Å for [CaCo] and [SrCo], and increases substantially for the Bi-materials, with n = 4: 14.65(1) Å for [BiCaCo], 14.89(2) Å for [BiSrCo], and 15.44(1) Å for [BiBaCo].

The Bi/Ba/Co ratio was studied by Energy-Dispersive X-ray Spectroscopy (EDS) on different points of the surface of each film. The amount of Sr was impossible to determine due to the interference with the lines of the Al coming from the LAO substrate (see supporting information Figure S3). For the other ions (Bi, Ba and Ca), deviations from the expected composition, according to the nominal concentration of ions in the starting solutions is very small (<10%), and within the accuracy limit of the technique given the thickness of the films (see supporting information Table S1).

On the other hand, the layered nature of the films will relax the epitaxial stress very fast. Therefore, a comparison between the c-axis lattice parameter of the films and single crystals is justified. This lattice parameter is very sensitive to the chemical composition^23^,^24^,^25^,^26^, and then it is helpful to further refine the stoichiometry obtained from EDS. By combining these two methods,the final stoichiometry of the films was estimated as: [Ca_2_CoO_3_][CoO_2_]_1.62_, [Sr_2_CoO_3_][CoO_2_]_1.8_, [Bi_1.68_Ca_2_O_4_][CoO_2_]_1.69_, [Bi_1.74_Sr_2_O_4_][CoO_2_]_1.82_, and [Bi_2_Ba_1.8_Co_0.2_O_4_][CoO_2_]_2_.

**Figure 2:**
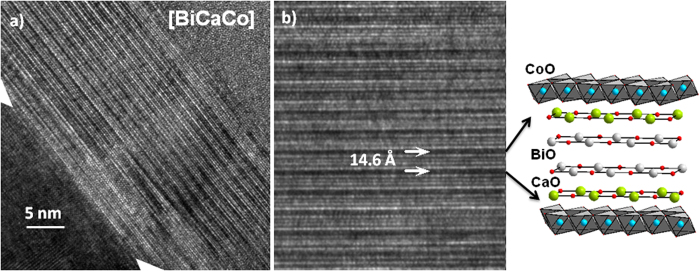
**a**) HR-TEM image of a ≈25 nm thick film of [BiCaCo]. **b**) Higher resolution detail of the structure showing the perfect layered assembly of the RS and CoO_2_ blocks, compared to the crystal model.

**Figure 3:**
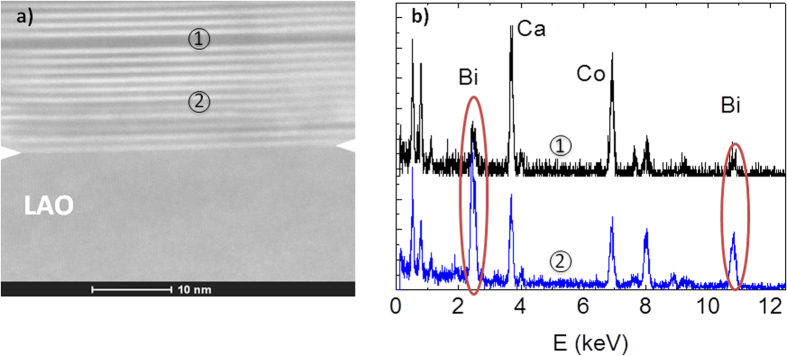
**a**) HAADF-STEM of the [BiCaCo] film. There is a clear contrast between the Bi-rich RS and Bi-poor planes. The image shows the regular stacking close to the interface with the substrate. **b**) EDS analysis at point 1 and 2, confirming the Bi rich (poor) composition of the bright (dark) layers.

The microstructure and the epitaxial relationship to the substrate was studied by high-resolution transmission electron microscopy (HR-TEM) in cross-section lamellae. The results for the [BiCaCo] are shown in Fig. 2. The different layers of the structure are clearly observed with a well-ordered growth along the c-axis. The structural repeating unit (see Fig. 2b) shows a distance of 14.6 (3) Å, in agreement with the out-of-plane lattice parameter obtained by XRD. We want to highlight that the interface with the substrate shows the absence of any amorphous layer (see also Figure S4 in the supporting information), showing a crystalline quality perfectly homogeneous across the whole film. The abruptness of the interface is also observed in the high angle annular dark field scanning transmission electron microscopy (HAADF-STEM, Fig. 3). In this image, the presence of stacking faults in some regions of the films is appreciated.

From the sensitivity of HAADF-STEM to the atomic mass of the constituent elements it is deduced that these defects in the self-assembled growth along the c-axis correspond to intergrowths with different composition. Indeed, EDS analysis of the layers with different contrast in the lamellae revealed a Bi-poor composition of the darker regions (Fig. 3b). These defects are also observed in thin films synthesized for physical methods and in single crystals^27^, and are characteristic of these misfit Co-oxides.

From the HAADF-STEM contrast and EDS profile analysis, it can be observed that the interfacial layer of the film in contact with the substrate is always the RS layer. This observation is crucial to explain the influence of the substrate in the crystalline quality of the sample.

**Figure 4:**
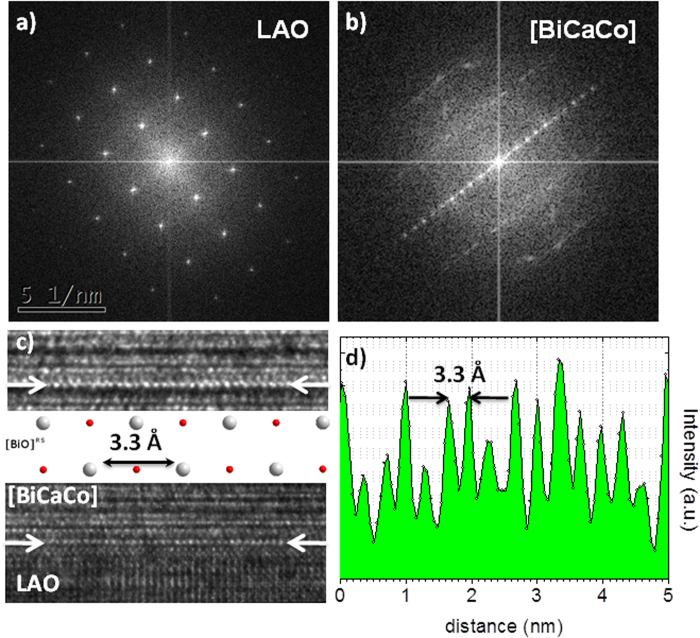
**a**) Fourier transform (FT) of LAO substrate showing the [100] orientation in the Pm-3m space group. **b**) The corresponding FT for the [BiCaCo] layer, verifying the epitaxial growth of the film. **c**) HR-TEM showing the ordered chains of atoms along a pseudocubic-layer of the RS block in the interface (bottom) and away from it (top). The distance is consistent with the Bi-Bi distance along the [110] direction of Bi_2_O_3_ (white circles in the structural model), as obtained from the EELS profile analysis in **d**).

The epitaxial growth of pseudo-cuadratic rock-salt layers of BiO and CaO (Fm-3m) on top of pseudo-cubic LAO (≈3.79 Å), will require an in-plane rotation of 45° in order to minimize the epitaxial strain. In this situation, the tensile strain imposed by the substrate will be ≈5.7%, which can be perfectly accommodated by the lattice^28^. The profile analysis along the RS layer (Fig. 4c) and d) confirms a Bi-Bi distance of ≈3.3 Å, as expected for the (110) plane of a pseudo-quadratic rock-salt layer of BiO. Therefore, the rock-salt layer grows epitaxially on top of the LAO substrate, with a relationship (001)BiO^RS^||(001)LAO; [110]BiO^RS^ ||[100]LAO. The analyses confirm that the orientation of the RS layers is maintained along the thickness of the film. Therefore, the almost perfect self-assembled structure observed along the c-axis is determined by the epitaxial growth of the first RS layer on top of the LAO substrate.

It is important to realize that this mechanism requires a very delicate accommodation of the first atomic layer, and consequently of quasi-equilibrium growth conditions. These are far from the growth mode in PLD or sputtering, and therefore slow-growth methods like this one are more adequate for epitaxial growth of chemically and structurally complex films.

**Figure 5:**
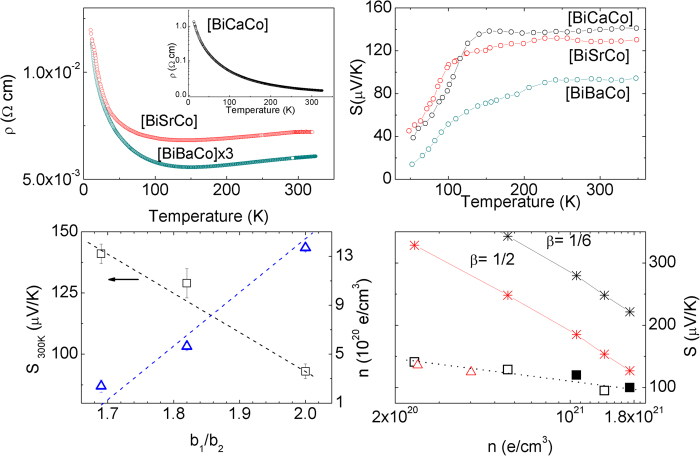
Temperature dependence of the electrical resistivity (**a**) and thermoelectric power (**b**) of the 4-layer Bi-compounds. **c**) Evolution of the Hall carrier density and S(300 K) with the misfit ratio. **d**) Evolution of S(300 K) with the Hall carrier density. Open triangles correspond to n = 3 compounds, and the squares to n = 4. The closed squares are taken from reference [41]. The asterisks mark the expected value of S on the basis of equation (1), for different values of the electronic degeneracy.

The transport properties of the 3-layer [CaCo] and [SrCo] are presented in Figure S5 in the supporting information. Their behavior is similar to the corresponding stoichiometric bulk samples^3^,^7^,^29^. Therefore, we will focus the discussion on the thermoelectric properties of the n = 4 Bi-films, as they offer the greatest novelty. The results are summarized in Fig. 5.

The electrical resistivity (Fig. 5a) is similar to single crystals, with a reentrant semiconducting behavior in the low temperature metallic phase of [BiBaCo] and [BiSrCo], and a semiconducting behavior for [BiCaCo] in the whole temperature range. There is a marked decrease in the electrical resistivity with the size of the cations at the RS layer, and therefore with the misfit ratio (b_1_/b_2_). This was previously observed in single crystals and associated to an intrinsic doping to keep the electrical neutrality. We have corroborated this hypothesis through an accurate determination of the density of charge carriers by Hall effect measurements (Fig. 5c).

On the other hand, the thermal stability of the films was probed by measuring the resistivity of the samples before and after repeated thermal cycles from room temperature up to 500 °C in air. The results for [BiSrCo] and [SrCo] thin films are shown in Figure S6, and show practically no changes after this thermal cycling.

The temperature dependence of the Seebeck coefficient, S, is shown in Fig. 5b. After a rapid increase, it saturates to a constant value above ≈150–250 K. This temperature independent thermoelectric voltage is typical of systems with localized, interacting carriers. In this case S is determined by the different possible distributions of holes (Co^4+^) in the available sites, i.e., the entropy per carrier^30^,^31^,^32^:


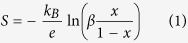


where β is the degeneracy of the electronic states of Co^3+^ and Co^4+^, and x is the concentration of holes.

The origin of the large S in the high temperature limit of these cobaltates is still the subject of an intense debate, and it is linked to the complex band structure of these materials^33^.

The trigonal distortion of the CoO_6_ octahedra breaks the orbital degeneracy of the band derived from the t_2g_ states, creating a wide in-plane e’_g_ band, and a narrower out of plane band with a_1g_ symmetry, which provides the electronic states at E_F_^34^. In the literature of the misfit compounds the degeneracy of Co^4+^ is either considered as 6 or 2, depending on the splitting of the energy levels^32^,^35^,^36^. However Photoemission experiments proved that the e’_g_-a_1g_ splitting is small compared to the bandwidth (at least in [BiCaCo]), and therefore is not able to reduce the spin degeneracy of Co^4+^ from 6 (t_2g_^5^) to 2 (e’_g_^4^ a_1g_^1^)^37^.

The experimental values of S in the temperature independent regime (>200 K) are shown in Fig. 5d) as a function of the actual concentration of holes measured by Hall effect. The thermopower decreases with n, but the slope is much smaller than expected from equation (1), independent of the value of β. Using the values of x obtained from ARPES^35^ (much larger than from Hall effect) also overestimates the experimental data. Therefore, there is an apparent discrepancy between the density of charge carriers measured by Hall, and the high temperature limiting value of S derived from equation (1).

On the other hand, UPS spectra of [BiSrCo] show a well defined Fermi edge at low temperatures, which broadens with increasing temperature (above ≈ 100 K)^35^. In less conductive [CaCo], this Fermi edge cannot be defined even at low temperature. Also, the band structure determined by ARPES shows a continuous transfer of spectral weight from a well defined quasiparticle peak (QPP) at E_F_, to a band of incoherent excitations (IEB) on approaching the band insulator regime from the itinerant side^34^. This occurs in the 4-layer compounds on going from itinerant [BiBaCo] to more localized [BiSrCo] and [BiCaCo]. The Hall effect also shows a characteristic temperature dependence^38^,^39^, which points to a charge density, effective mass, and scattering time consistent with the formation of small polarons. These are evidences that suggest that the incoherent motion of charge carriers progressively dominates the electronic transport as temperature increases.

Given that hopping transport does not contribute to Hall effect^40^, but the statistical distribution of these carriers will dominate the high temperature Seebeck coefficient through equation (1), the discrepancy between the Hall and Seebeck charge densities should be larger for the more localized samples, and should progressively approach each other in the more itinerant samples, as observed in Fig. 5d).

Therefore, the incoherent motion of carriers becomes the dominant contribution to the electronic transport, due to the temperature dependent transfer of spectral weight between the QP and the IEB. If this is the most probable explanation for the discrepancy between the carrier density determined by Seebeck and Hall data, obtaining information about the electronic degeneracy (β = 1/6 vs 1/2) from the experimental S is unreliable, unless an independent measurement of the concentration and the size of the polarons is available.

In summary, using an environmentally friendly and scalable chemical route, we have been able to overcome the difficulties in the synthesis of thin films of 3-layer and 4-layer misfit cobalt oxides. The possibility of fabricating thin films of thermoelectric Co-oxides with the quality reported here, free of amorphous interfacial layers, opens the possibility of integration of these materials with complementary n-type thermoelectric oxides in “all-oxide” thermoelectric modules.

Finally, on the basis of accurate thermoelectric measurements the apparent discrepancy between Seebeck and Hall data is explained, considering the charge redistribution among the polaronic and itinerant bands of these compounds.

## Additional Information

**How to cite this article**: Rivas-Murias, B. *et al.* High quality thin films of thermoelectric misfit cobalt oxides prepared by a chemical solution method. *Sci. Rep.* **5**, 11889; doi: 10.1038/srep11889 (2015).

## Supplementary Material

Supplementary Information
