# Supplementary material for: High quality thin films of thermoelectric misfit cobalt oxides prepared by a chemical solution method
Source: Sci Rep. 2015 Jul 8;5:11889. doi: 10.1038/srep11889 (PMC4495387; doi:10.1038/srep11889)
Supplement: Supplementary Information [file srep11889-s1.pdf]

Supporting Information for:

High-quality thin films of thermoelectric misfit cobalt oxides prepared by a chemical solution method.

*B. Rivas-Murias, J. M. Vila-Fungueiriño, F. Rivadulla**

Centro de Investigación en Química Biológica y Materiales Moleculares (CIQUS), Universidad de Santiago de Compostela, 15782-Santiago de Compostela, Spain.

Table S1: Comparison of the nominal (starting solution) and experimental compositions. The amount of Sr could not be determined by EDS due to interference with Al from $LaAlO_3$.

	Nominal composition	Experimental (EDS)
[BiBaCo]	Bi:Ba=1.11	Bi:Ba=1.12(3)
	Bi:Co=0.91	Bi:Co=0.85(4)
[BiSrCo]	Bi:Sr=1	Bi:Sr= --
	Bi:Co=1	Bi:Co=1.04(3)
[BiCaCo]	Bi:Ca=0.84	Bi:Ca=0.87(2)
	Bi:Co=1	Bi:Co=0.98(2)
[CaCo]	Ca:Co=0.75	Ca:Co=0.69(3)
[SrCo]	Sr:Co=0.75	Sr:Co= --

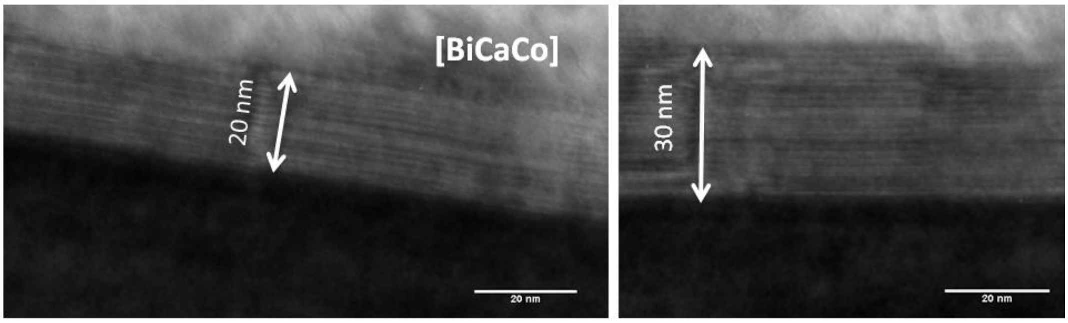

Figure S1: TEM images of different cross-section lamellae of [BiCaCo]. The thickness of the films is indicated in each panel.

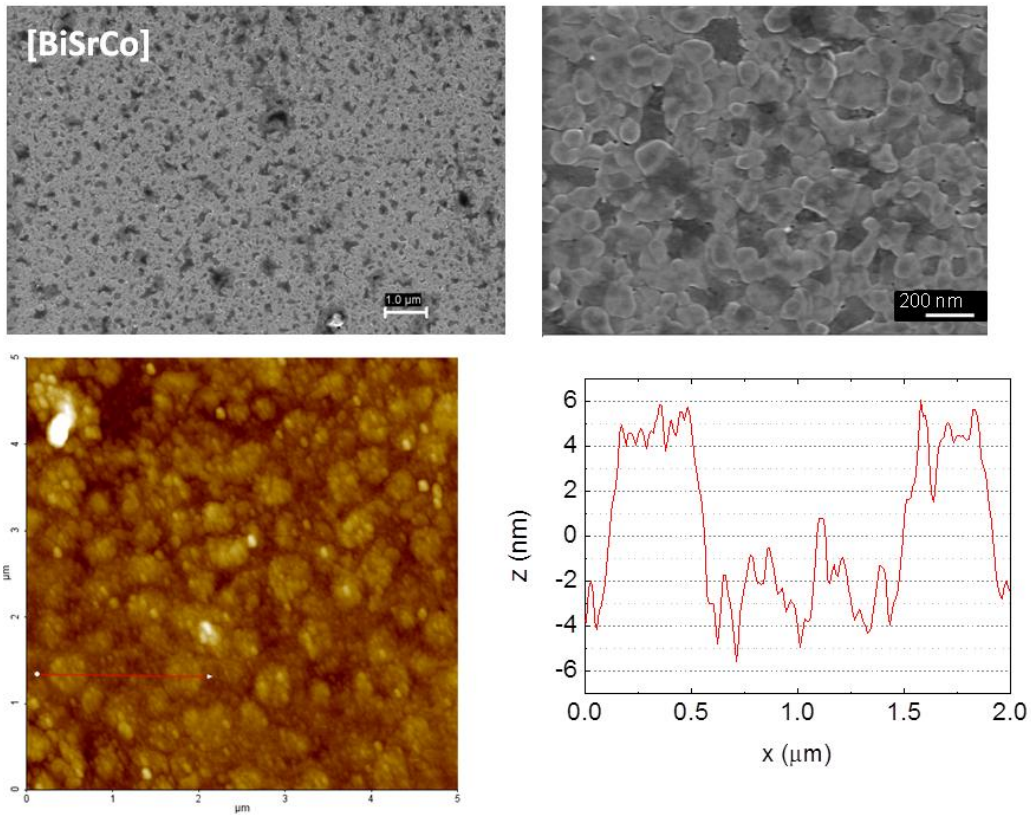

Figure S2: Top: SEM images of the surface of a [BiSrCo] thin film, under different magnifications. Bottom: 5x5 μm^2 AFM image of the surface of the film. The flat platelets can be appreciated in the and height profile.

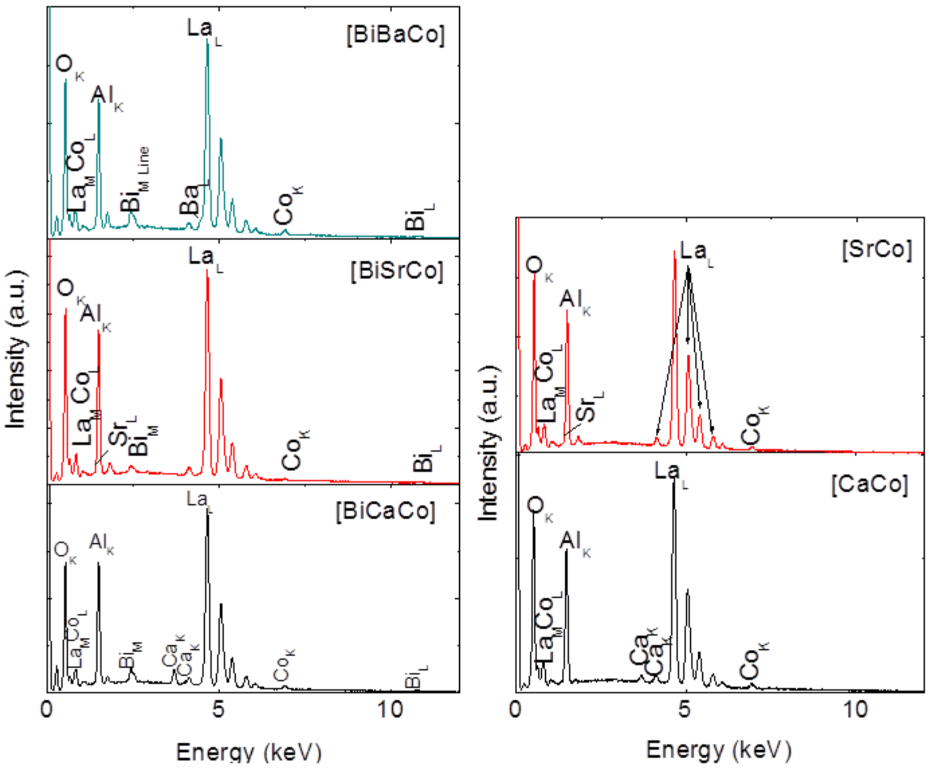

Figure S3: Example of EDS analysis for the different films studied in this work.

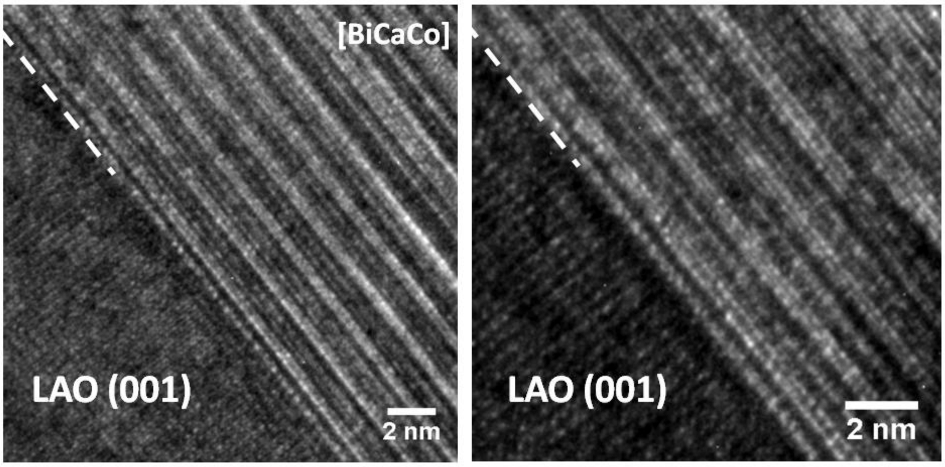

Figure S4: Detail of the interface of [BiCaCo] films.

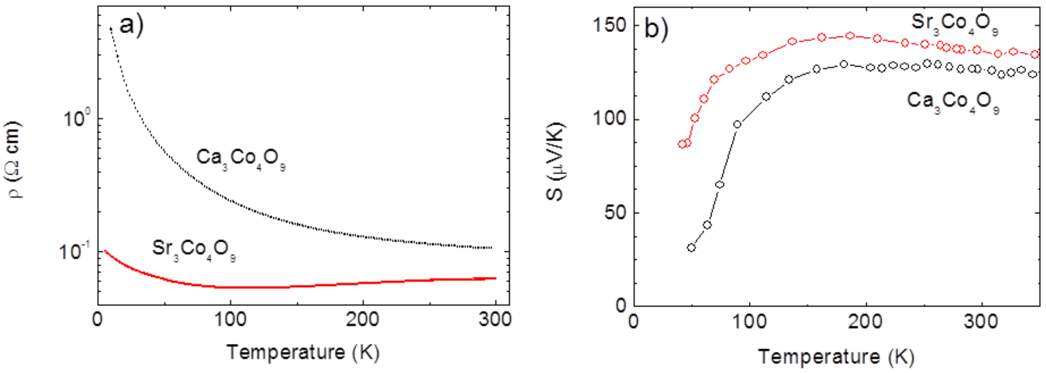

Figure S5: Temperature dependence of the electrical resistivity (a) and Seebeck coefficient (b) for the 3-layer compounds $Ca_3Co_4O_9$ and $Sr_3Co_4O_9$.

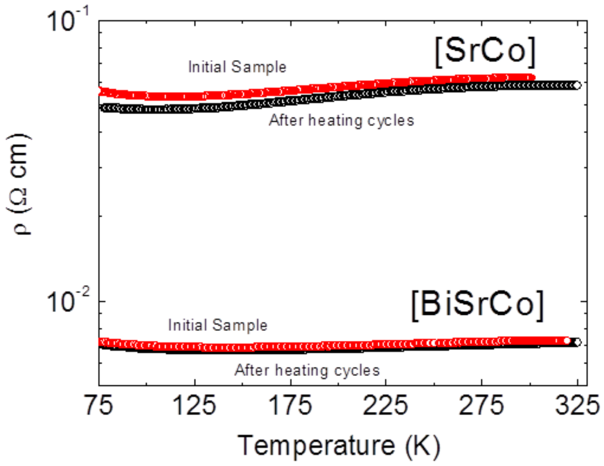

Figure S6: Temperature dependence of the electrical resistivity of [SrCo] and [BiSrCo] thin films, as prepared, and after being cycled between room temperature and 500ºC (in air), three times.
